# Advancements in Inorganic Membrane Filtration Coupled with Advanced Oxidation Processes for Wastewater Treatment

**DOI:** 10.3390/molecules29174267

**Published:** 2024-09-09

**Authors:** Chaoying Zhang, Rongfang Yuan, Huilun Chen, Beihai Zhou, Zexin Cui, Boyun Zhu

**Affiliations:** Beijing Key Laboratory of Resource-Oriented Treatment of Industrial Pollutants, School of Energy and Environmental Engineering, University of Science and Technology Beijing, Beijing 100083, Chinazhoubeihai@sina.com (B.Z.);

**Keywords:** ceramic membranes, carbon-based membranes, membrane filtration, AOPs, wastewater treatment

## Abstract

Membrane filtration is an effective water recycling and purification technology to remove various pollutants in water. Inorganic membrane filtration (IMF) technology has received widespread attention because of its unique high temperature and corrosion resistance. Commonly used inorganic membranes include ceramic membranes and carbon-based membranes. As novel catalytic inorganic membrane processes, IMF coupled with advanced oxidation processes (AOPs), can realize the separation and in situ degradation of pollutants, thus mitigating membrane contamination. In this paper, the types and performance of IMF are discussed. The influencing factors of inorganic membranes in practical wastewater treatment are summarized. The applications, advantages, and disadvantages of the coupled process of IMF and AOPs are summarized and outlined. Finally, the challenges and prospects of IMF and IMF coupled with AOPs are presented, respectively. This contributes to the design and development of coupled systems of membrane filtration with inorganic materials and IMF coupled with AOPs for practical wastewater treatment.

## 1. Introduction

With the rapid growth and development of the global economy, large quantities of wastewater with organic pollutants are discharged into natural water through industrial and residential drainage systems every day (for example, 36,000 t/year in China) [1]. Most organic compounds have been covered by monitoring systems, but emerging pollutants were only discovered over a decade ago. This class includes personal care products, pharmaceuticals, pesticides, etc. Due to the increase in sewage discharge and the emergence of emerging pollutants, they pose serious threats to the environment and human health. [2]. To protect water resources, measures need to be taken to remove pollutants from wastewater before they are discharged into the ecosystem. Processes commonly used at this stage include adsorption, coagulation, membrane filtration, advanced oxidation processes (AOPs), and combined processes [3,4,5]. Among them, membrane filtration is considered to be an effective water recovery and purification technology that can remove various pollutants from water [6,7].

Membranes for membrane filtration can be divided into inorganic membranes, organic membranes, and organic–inorganic hybrid composite membranes based on the constituent materials [8]. Organic membranes are usually made of polymeric materials (such as cellulose acetate, polyethersulfone, polyfluoropolymers, etc.) [9,10]. Organic membranes are less expensive. During membrane filtration, the membrane is prone to blockage and contamination, requiring regular cleaning [11,12]. However, the mechanical strength and long-term stability of organic membranes are poor, which shortens their lifespan and requires regular replacement, increasing operating costs [2,13]. Bacteria present in the water can degrade organic membranes, whereas inorganic membranes are less susceptible to bacteria [14]. Compared with organic membranes, inorganic membranes have the characteristics of lower oxidation resistance, higher mechanical strength, higher hydrophilicity, more antifouling properties, more acid and alkali resistance, and higher temperature resistance [14,15,16,17]. Inorganic membranes can be applied in extreme environments, so they have received increasing attention. Membrane fouling and subsequent treatment of membrane filtration concentrate are the core issues in the membrane filtration process.

AOPs mainly include photocatalytic oxidation, Fenton oxidation, ozonation, persulfate oxidation, etc. [18,19,20]. In contrast to the physical process of membrane filtration, AOPs can utilize reactive substances generated by chemical oxidation to degrade and mineralize organic compounds [21,22]. With the help of catalysts, the technology can generate highly reactive free radicals faster and remove pollutants more quickly and completely [23]. However, in the process, nano-catalysts are often more effective, but they are difficult to fully recover [24]. Post-treatment is required after the reaction to recover the catalyst [25]. The coupling of membrane separation and AOP exhibits the synergistic effect of chemical reaction and physical separation. Membrane filtration coupled with AOPs has been widely studied in recent years.

Membrane filtration coupled with AOPs is the preparation of catalytic membranes by fixing catalyst particles onto the membrane surface or doping them onto the membrane skeleton. Catalytic membranes can avoid the agglomeration of catalysts and obtain the ability to oxidize and degrade pollutants while separating them from water [26]. The catalytic membrane not only provides effective recovery of powdered catalyst, but also enhances the mass transfer in the catalytic oxidation process [27], improving the efficiency of organic matter removal. On the other hand, the process achieves simultaneous catalytic and membrane separation, which can improve the anti-fouling performance of the membrane. Therefore, inorganic membrane filtration (IMF) coupled with AOPs is a water treatment process that can be operated continuously [28]. In practical applications, various coupling processes still face various problems, and this combination system needs further improvement.

So far, there have been numerous reviews on the preparation methods and performance of separation membranes, and the mechanism and application of AOPs, respectively. However, there have been few comprehensive analyses of membrane filtration of inorganic materials and the coupling process of IMF and AOPs. In this paper, the types and properties of inorganic membranes and the influencing factors of IMF in practical wastewater treatment are first summarized. Then, the application, advantages, and disadvantages of new catalytic IMF processes (including IMF-photocatalytic oxidation, IMF-ozonation, IMF-persulfate oxidation, and IMF-Fenton oxidation) are generalized. Finally, the challenges and future development prospects faced by the IMF and the coupled processes of IMF and AOPs are presented. The growth of membrane technology relies on developing new materials and membrane modification processes. The main research direction in the future is to develop high-throughput, high-strength, long-life, pollution resistant, and low-cost membranes.

## 2. Inorganic Membrane Filtration

Membrane filtration is a low technology process that utilizes the selective permeability of a membrane and applies pressure to the membrane to allow specific components to pass through and separate [29]. Based on the pore size of the membrane, pressure-driven membrane technologies can be categorized into four types: microfiltration (MF), ultrafiltration (UF), nanofiltration (NF), and reverse osmosis (RO). The main types of inorganic membranes include dynamic membranes, liquid membranes, silica, zeolite, carbon-based membranes (CBMs), and ceramic membranes (CMs) [30]. The two most widely used inorganic membranes for wastewater treatment, CMs and CBMs [31], have been used in a large number of applications in industrial wastewater.

### 2.1. Inorganic Membrane Types

#### 2.1.1. Ceramic Membranes

**Figure 1:**
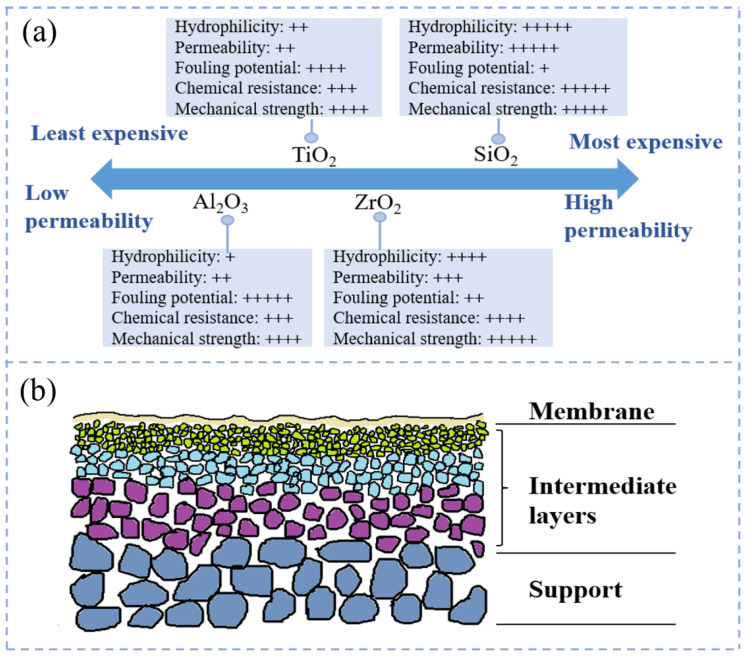
(**a**) CM performance characteristics of different materials used for wastewater treatment [31]; (**b**) schematic of CM cross-section.

CMs are generally prepared from chemically resistant metal oxides (Al_2_O_3_, TiO_2_, SiO_2_, ZrO_2_, etc.) or their mixtures, and are currently the most widely used inorganic films on the market [32]. Among them, Al_2_O_3_ is a widely used ceramic material for membrane fabrication. Al_2_O_3_ membranes have high strength, chemical stability, and thermal stability, and are more economical and cost-effective [31,33]. ZrO_2_ membranes have a monoclinic crystalline structure at room temperature and tetragonal and cubic structure at high pressure [33]. Due to their remarkable hydrophilicity and excellent heat resistance, they are suitable for oil–water separation [34,35]. TiO_2_ membranes contain rutile, anatase, and limonite, and have anti-fouling and anti-bacterial properties [34]. SiO_2_ membranes have smaller pore sizes, are less likely to be clogged during use, and can be applied to seawater desalination [30]. Ceramic membrane materials are listed according to their chemical stability from the highest strength to the lowest as follows: TiO_2_ > ZrO_2_ > Al_2_O_3_ > SiO_2_ [34]. The relative properties of the aluminum (Al)-, zirconium (Zr)-, silicon (Si)-, and titanium (Ti)-based ceramic membranes are presented in Figure 1a [31]. The structure of CMs is an asymmetric structure composed of multiple layers of oxides with different pore sizes and porosities. The asymmetric structure generally has 2–3 layers, including a support layer, an optional intermediate layer, and an active layer (Figure 1b). The support layer is prepared from adhesives and gelling agents, and the support layer provides mechanical support for the membrane as well as reduces the solvent permeation resistance [36,37]. The intermediate layer is thin and porous with a pore size distribution in the range of 50–500 nm, functioning to reduce the pore size and produce a smooth membrane surface [38]. The active layer, with a thickness of less than 1 μm, determines the membrane’s porosity and is used to fulfill the separation function of the membrane [13,39].

The steps involved in the preparation of CMs are as follows: particle suspension formation, membrane molding, membrane sintering, and precision processing [37]. Membrane molding can be further classified into slip casting, tape casting, the pressing method, freeze-casting, and direct foaming [40,41,42,43,44]. Among these, pressing is considered to be the simplest method of manufacturing CMs. In this step, the ceramic material is shaped into the desired geometry. Typically, CMs come in two main shapes, flat and tubular, and both have multiple channels. Tubular CMs have a higher specific surface area [45]. The sintering step is crucial for preparing a membrane with good mechanical strength and narrow pore size distribution. The membrane sintering process consists of three key steps, re-sintering, thermolysis, and final sintering [46]. The sintered membrane develops final mechanical strength and controls grain growth, relative density, shrinkage, and morphology. The final step is to change the membrane hydrophilicity, pore size, and surface roughness by surface modification. The physical methods involved include surface coating, layer-by-layer self-assembly, etc. [47,48]. Chemical methods include immersion, chemical vapor deposition, sol-gel, in situ reduction, etc. [37,46].

The CMs of MF and UF are widely used in surface water treatment and wastewater treatment to remove possible dissolved organic matter, suspended matter, heavy metals, bacteria, etc. [49,50,51]. Most CMs are separated based on the size exclusion of membrane pores and the electrostatic effect on the membrane surface [52]. Depending on the particle size, effective rejection (>90%) of suspended particles and bacteria could be achieved by membranes with pore sizes of >10 nm, 10–30 nm, and 10–1100 nm, respectively [53]. MF can serve as a means of pretreatment to ensure the stable operation of subsequent processes. Zhong [54] used ZrO_2_ membrane for MF treatment of oily wastewater generated in refineries and observed that CM exhibited better separation performance and a higher oil removal rate than organic membrane. Cui et al. [55] used a 50 nm ZrO_2_/Al_2_O_3_ membrane for pretreatment of seawater desalination, and the CM system was able to maintain long-term stable permeability under low-temperature (3–6 °C) conditions. Kim et al. [56] observed that ceramic UF membranes with pore sizes less than 4.0 nm can trap surfactant sodium dodecylbenzene sulfonate (SDBS). At high TMP, the substances formed by SDBS on the membrane surface caused concentration polarization, which induced a prescreening effect, increasing the retention of SDBS. The electrostatic repulsion between the surface charge of the membrane and the charged particles in the solution causes solute retention, and changes in pH value can affect the retention rate of the target substance [57]. CMs in membrane bioreactors (MBRs) allow the reactor to operate at high mixed liquor suspended solids (MLSSs) concentrations and high fluxes, so CMs can be used for larger scale wastewater treatment. In a recent study [51], the removal of micropollutants by CMBR was evaluated, with the antibiotic ofloxacin removing 98%.

The challenge of using CM can be summarized as reducing the cost of CM by reducing the number of steps in the fabrication process and lowering the sintering temperature. Metal-organic framework (MOF) membranes can be prepared at lower temperatures [14]. MOF membranes can be supported on polymeric membranes and CMs to prepare NF membranes with high selectivity [58]. Yuan et al. [59] loaded zeolite imidazolate framework (ZIF) on the substrate α-Al_2_O_3_ to prepare a novel pure ZIF-300 membrane for the removal of heavy metal ions from the aqueous environment. The prepared membranes showed a retention rate of 99.21% of CuSO_4_ and improved water permeability (39.2 L m^−2^·h^−1^·bar^−1^). The ZIF-300 membranes exhibited excellent water stability and particle size discrimination, but the affinity between the MOF layer and the ceramic substrate is low, so producing a sturdy ceramic MOF film is still a big problem. Zeolite, fly ash, kaolinite, and mullite can be used as raw material substitutes for the preparation of CMs [37,60], which can also reduce the cost. It is worth noting that commercial NF membranes of ceramics are difficult to mass produce because NFs require hard textured support and intermediate layers, and the preparation of defect-free NF membranes has high process requirements. In the future, it is necessary to further develop CMs with low cost, a long lifespan, high selectivity, and strong antifouling performance. The new membrane materials generated by the hybridization of inorganic membranes and polymer membranes are also a new research direction.

#### 2.1.2. Carbon-Based Membranes

The emergence of several new materials, especially carbon-based nanostructures such as carbon nanotubes (CNTs), graphene, and their derivatives, has provided a new era in enabling membrane science and technology with separation properties [61]. These materials have important properties such as a large specific surface area, high permeability, homogeneous structure, tunable pore size, and strong atomic bonds [62], and thus have attracted increasing attention in solving water pollution and water scarcity problems.

(1) Carbon nanotube membranes

According to the different layers and shells, CNTs can be further divided into single-walled carbon nanotubes (SWCNTs), double-walled carbon nanotubes (DWCNTs), and multi-walled carbon nanotubes (MWCNTs), which have smooth internal hydrophobic surfaces [63]. CNT membranes are prepared by incorporating CNTs into polymer matrices [61,64], and CNTs are also arranged in vertical support fillers such as epoxy resin and silicon nitride. CNT membranes are usually classified into vertically aligned CNT membranes, horizontally aligned CNT membranes, and mixed-matrix CNT membranes [64]. The proper pore size of well-arranged CNT membranes can screen contaminants of a certain size, reject salt ions, and allow water to pass through the pores [65]. Meanwhile, the hollow CNT structure provides frictionless transport of water molecules to enhance water permeability and provides excellent water flux for simple functionalization [66]. 

Vertically aligned CNT membranes have high water flux, which was demonstrated by Baek et al. [67] to be up to three times higher than that of typical ultrafiltration membranes. It has been reported that DWCNT membranes with pore sizes below 2 nm exhibit extremely fast water flow rates of up to 6 × 10^3^ L cm^−2^ day^−1^ MPa^−1^, which is two orders of magnitude greater than that of reverse osmosis membranes (~2.6 × 10^2^ L cm^−2^ day^−1^ MPa^−1^) [68]. Sadia et al. [69] studied membranes made solely from CNTs. In the experiment, H_2_O_2_ oxidants were added, and the phenol removal rate of this membrane exceeded 85% within 4 h, with an average oxidation rate of about 0.059 mol.h^−1^.m^−2^. Water molecules can be transported through CNT structures without significant impedance, thus developing some CNT membranes with excellent desalination rates and high permeability for RO systems [70,71]. Chen et al. [72] designed an asymmetric tip-functionalized CNT membrane, with one tip containing a hydrophilic group (carboxyl group) and the other tip containing a hydrophobic group (trifluoromethyl group), effectively blocking salt ions with pore sizes of 0.81 nm and 1.09 nm. Corry et al. [73] placed a series of functional groups with different charges and polarities on the tip of CNTs with a diameter of 1.1 nm. They found that eight carboxyl groups with negative charges prevented the passage of Na^+^ and Cl^−^ and were able to achieve a desalination rate of 100%. Currently, research has found that modified CNTs can achieve better selectivity [74]. Zhang et al. doped the CNT-based hybrid film with Au nanoparticles. The prepared film has the multi-function of circulating catalytic degradation of soluble organic molecules and separation of oil–water lotion. The maximum flux is up to 3000 L m^−2^·h^−1^·bar^−1^. It decomposed 92.6% nitrophenol in oily wastewater.

(2) Graphene membranes

Graphene is a material composed of the compact accumulation of sp2 hybridized carbon atoms. Graphene and graphene oxide (GO) have been widely used to construct novel membranes with layered pores [75]. Since the flux through a membrane is known to be inversely proportional to the thickness of the membrane, graphene nanosheets with single-atom thickness and two-dimensional structure offer great promise for high-throughput and energy-efficient separations [76]. Graphene materials have been recognized as the new generation of RO films, which have the advantages of a smooth surface, low roughness, few nucleation sites, and low adhesion of scaling crystals. It is more robust, thinner, chemically stronger, and ion-selective than the active layer in polymeric RO membranes [77]. Kabiri et al. [78] synthesized a thiol-functionalized graphene membrane with a unique three-dimensional porous structure for the removal of mercury ions (Hg^2+^) from water. The results showed that the removal rate was almost 100% for low (4 mg/L) and high (120 mg/L) concentrations of Hg^2+^. Graphene membranes have a size exclusion effect. O’Hern et al. [79] reported that they controlled the formation of high-density sub-nanopores on graphene membranes, which transported salts and removed larger organic molecules. While graphene oxide (GO) is prepared by oxidizing graphite with strong acids or oxidants and has hydroxyl, epoxide, and carboxyl groups [80], GO has better water dispersibility than graphene and can be well dispersed in water and other organic solvents. Good water dispersibility is beneficial for preparing GO-based membranes [81,82]. Li et al. [83] prepared porous Al_2_O_3_ tube-loaded GO composite membranes with sub-micrometer thickness and improved mechanical strength using a press-filtration deposition method, which can achieve efficient dehydration of organic solvents. Zhao et al. [84] prepared a GO membrane with adjustable interlayer spacing using a simple thermal reduction method. The prepared membrane had relatively high permeability (17.1 LMH/bar) and a high removal rate for heavy metal ions (i.e., the removal rates for Cu^2+^, Pb^2+^, Cd^2+^, and Ni^2+^ are 98.6%, 97.2%, 99.1%, and 97.2%, respectively). Chang et al. [85] reported that carboxylation could enhance the hydrophilicity of GO membranes, thereby improving dye removal efficiency. Nowadays, GO membranes are also commonly used to remove oil from wastewater. Gao et al. [86] made a pressure-driven membrane from sulfonated graphene oxide (SGO) nanosheets and hierarchical nanostructured TiO_2_ spheres. The flexible and super hydrophilic SGO-TiO_2_ membrane can effectively separate oil–water lotion stabilized by surfactants.

Despite the rapid growth and development of CNT-related research at the laboratory scale, the commercial application of CNT membranes in water treatment has proceeded at a slow pace, mainly due to the high production cost of high-quality and reproducible carbon nanotubes. Common CBMs also include coal-based carbon membranes, phenolic resin-based carbon membranes, and carbon fiber membranes. The low price of coal and phenolic resin can reduce the manufacturing cost of CBMs. Meanwhile, it was found that SWCNT was bio-persistent and could be observed to induce inflammation and lung cell proliferation in rat lungs. Risk assessment is crucial before applying these carbon nanomaterials.

### 2.2. Influences

The retention effect of IMF on contaminants varies considerably. The feed water conditions of the influent solution have an important influence on the retention process, making the retention effect different under different circumstances. Different inorganic membranes have widely varying properties in terms of pore size, hydrophilicity of the material, roughness, etc., and can lead to differences in contaminant removal. The presence of membrane contamination can complicate the retention process, and the retention rate may appear to increase or decrease.

#### 2.2.1. Coexisting Substances in Water

Coexisting substances in water include temperature, pH value, inorganic ion concentration, natural organic matter (NOM), filtration pressure, etc.

**Figure 2:**
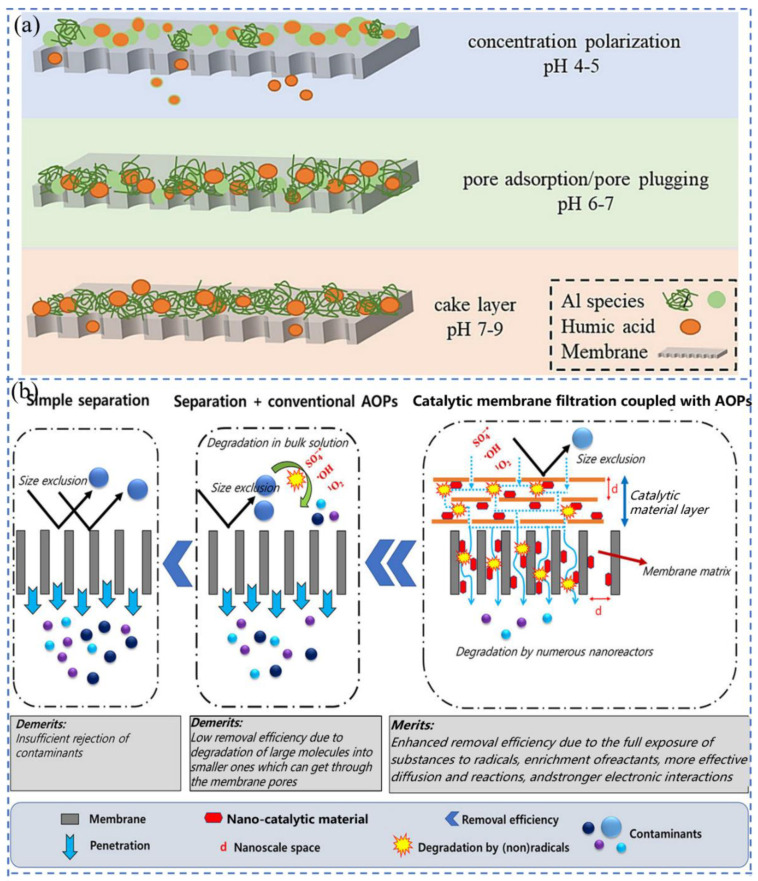
(**a**) The main membrane-fouling mechanisms for the three coagulants [88]; (**b**) schematic diagram of individual separation, separation and conventional AOPs, and catalytic membrane filtration coupled with AOPs [27].

Temperature is related to the permeate water viscosity, which affects pure water flux. Akhondi et al. [87] found that when the operating temperature was varied from 21 ± 1 °C to 29 ± 1 °C, the fouling resistance was reduced by 25% at a driving force of 40 mbar and 21% at 100 mbar. Changes in pH value can cause changes in the surface charge of NF membranes as well as dissociation of target substances, altering the electrostatic interaction between the membrane and the target substance, thereby altering the retention rate of the target substance. In Figure 2a, Wang et al. [88] studied the effect of different pH values on the retention efficiency of HA. When pH value is lower, the size of pollutants is smaller, the electrostatic force between the surface charges of the film is weakened, and the hydrophobicity of the film is increased. At pH 4, the flocs formed had a small particle size and were prone to membrane clogging due to concentration polarization. Under neutral conditions, the floc structure was loose, and the main cause of membrane clogging was pore clogging. Kramer et al. [89] used ceramic NF membranes with a pore size of about 0.9 nm to retain phosphate. Phosphate was present in the form of H_2_PO_4_^−^ and HPO_4_^2−^ with hydrodynamic radii of 0.302 nm and 0.327 nm, respectively. Depending on the pore size of the membrane and the hydrodynamic radius of the phosphate, phosphate would not be retained. But in practice, the phosphate retention rate increased from 76% to 99% as the pH value increased in the range of 5–9. The addition of inorganic ions cannot increase the permeation flux, as salt can lead to an increase in viscosity in the pores and bulk solution [90]. 

The effect of the presence of NOM on contaminant retention is not well established. Zazouli et al. [91] used two commercial composite nanofiltration membranes (SR2 and SR3 membranes) with different properties. The SR2 membrane had a larger pore size and higher membrane flux. In the absence of NOM, the removal of PhACs by SR3 membranes was greater than that of SR2 membranes (except for cefadroxil). Alginate was used as a NOM model, and the removal of indomethacin and tetracycline increased in both SR2 and SR3 membranes when 25 mg/L alginate was added. And acetaminophen removal decreased. NOM was completely retained by the membrane and formed a fouling layer on the membrane surface, thereby increasing the permeate flow resistance [92]. The increased removal of both indomethacin and tetracycline can be explained because the alginate fouling layer not only offers charge repulsion between the carboxylic acid group of the drug and the alginate group, but also enhances size exclusion [93]. The log P of acetaminophen is moderate and will accumulate in the polar alginate layer, making it easier to diffuse to the permeate side through the membrane barrier, thereby reducing the interception effect. During filtration, Ca^2+^ and Mg^2+^ accumulate on the membrane surface until precipitation is formed [94,95]. Over time, these precipitates form microcrystals, leading to inorganic fouling on the nanofiltration membrane, which reduces the membrane flux [96]. Su et al. [97] titrated different concentrations of Al^3+^, Fe^3+^, and Cu^2+^ solutions in NOM samples. When the concentration of the titrated Cu^2+^ solution was 5 μm, its complexation ability with NOM was strong, which resulted in a decrease in the membrane flux. When the concentration of the titrated Al^3+^ and Fe^3+^ solution was 20 μm, the complexation ability of ions with NOM was relatively weak, and micro flocs would be formed to block the nanofiltration membrane. Laitinen et al. [98] investigated the UF-influencing factors of membrane filtration. At a cross-flow velocity of 3 m/s, the pressure increased from 0.7 bar to 2.3 bar, the flux increased by 22 LMH (23%), and the pressure–flux curve started to level off after 1 bar. This is because as the pressure increases, the fouling layer becomes thicker/denser, making it more resistant to osmotic flow. As the cross-flow velocity increases, the increase in flux becomes greater due to the reduction of the pollution layer.

#### 2.2.2. Inorganic Membrane Properties

The properties of inorganic membranes, such as pore size, surface roughness, hydrophilicity, or hydrophobicity, have a significant impact on membrane filtration performance.

Due to the size exclusion effect, filtration membranes are able to remove particulate matter larger than the membrane pore size [99]. Therefore, smaller pores exert a stronger interception effect [100]. The reduction of membrane pore size can improve the selectivity of the membrane and may prevent the occurrence of internal scaling. As the pore size of the membrane increases, the pure water flux of the membrane also increases. A higher pure water flux means an increase in membrane filtration rate, but a decrease in pollutant removal efficiency. In GDM filtration [101], the water flux of flat plate membranes with pore sizes of 0.22 and 0.45 μm was 227 LMH, and 679 LMH, respectively. Ideal separation membranes should have both good selectivity and high permeability. A membrane with high permeability reduces the required membrane area for water treatment, ultimately leading to a decrease in the cost of membrane filtration [63]. The roughness of the membrane determines the separation efficiency, as it affects the membrane’s anti-fouling properties [102]. Sun et al. [103] developed a smooth ceramic-based graphene seawater desalination membrane, confirming that graphene membranes exhibit more stable water flux and almost complete desalination rate (>99.9%) for high saltwater treatment. Hydrophilic membranes (water contact angle < 90°) can form a water molecule layer on the surface to prevent contact with contaminants in solution [61]. Different types of wastewater use membranes with different properties. For example, in oil–water separation, the membrane can be hydrophobic and oleophilic, or hydrophilic and oleophobic [104]. Hydrophobic membranes are preferred over hydrophilic membranes in membrane distillation [105]. Membranes can be modified to alter the membrane surface properties. Mallya et al. [106] used hydroxyl-functionalized molybdenum disulfide (OH-MoS_2_) nanosheets as nanofillers to design NF membranes. After 6 h of experiment, the retention rate of SA remained at 93%, while the control group only had 74%. The modified membrane had higher hydrophilicity, a negative charge, and rougher membrane morphology, which could support the formation of a water hydration layer, thus improving the antifouling and NOM removal performance. Coelho et al. [107] fabricated ceramic UF membranes with ZrO_2_ slurry dip-coated with porous SiC carriers; the membrane pure water permeation flux was up to 360 L/(m^2^·h·bar), and the pilot test of olive oil/water emulsion removed 99.91% of the oil.

Membrane contamination is complex, diverse, and unavoidable. Fouling is caused by the accumulation of organic and inorganic pollutants during the membrane filtration process, and the permeation flux decreases over time [21]. Four possible models of membrane fouling include complete pore blocking, intermediate pore blocking, cake filtration, and standard pore blocking [31]. In applications, to safeguard the effectiveness of membrane filtration, various influencing factors will be controlled as much as possible to provide a suitable environment, but this can only slow down the time for membrane contamination to occur. Pretreatment and cleaning of membranes can restore membrane flux [108,109,110,111,112,113]. Ghadimkhani et al. [114] added HA to the membrane unit system under alkaline conditions to simulate organic pollutants. After the membrane was completely scaled, air nanobubbles were introduced into the CM support for backwashing. After 6 h, the permeation flux of CM basically recovered to 99%. Although membrane cleaning can remove reversible fouling, it is still difficult to restore performance if irreversible membrane contamination occurs. Moreover, frequent cleaning operations will reduce the service life of the membrane and increase the operating cost. Scholars have found that inorganic membranes can be modified to improve their performance and reduce the occurrence of irreversible pollution [106,107,115,116]. The techniques for membrane contamination mitigation are summarized in Table 1 [107,114,117,118,119,120,121]. Materials that can provide high permeability, high selectivity, and low energy consumption will become the most effective materials for incorporating membranes. Using AOPs to degrade contaminants in wastewater before filtering improves removal efficiency but adds additional process steps [27]. However, membrane filtration coupled with AOPs can simultaneously degrade and remove pollutants (Figure 2b) [27], which accelerates mass transfer of organic pollutants and oxidants from the native solution to the catalyst surface, increasing the production rate of oxidized substances. This facilitates the interaction between the contaminants and the generated free radicals, thereby increasing the reaction rate and removal efficiency [122,123,124].

## 3. Inorganic Membrane Filtration Coupled with AOPs

The intercepted pollutants in membrane separation still exist, which restricts its application in water treatment. AOPs can be divided into hydroxyl radical (•OH)-based AOPs (HR AOPs) and sulfate radical ($SO_{4}^{-}•$)-based AOPs (SR AOPs). By coupling membrane filtration with AOPs technology and utilizing catalysts to produce highly oxidizing active substances, organic pollutants in water can be mineralized and decomposed in situ. Polymeric membranes cannot be exposed to free radicals for a long period, and prolonged contact with oxidizing agents will cause wear and tear and reduce the service life. Therefore, inorganic membranes are more suitable for coupling with AOPs [31,125]. Catalytic separation membranes will be a new generation of functional membrane products and have a promising application in future water treatment. 

### 3.1. IMF Coupled with Photocatalytic Oxidation System

**Figure 3:**
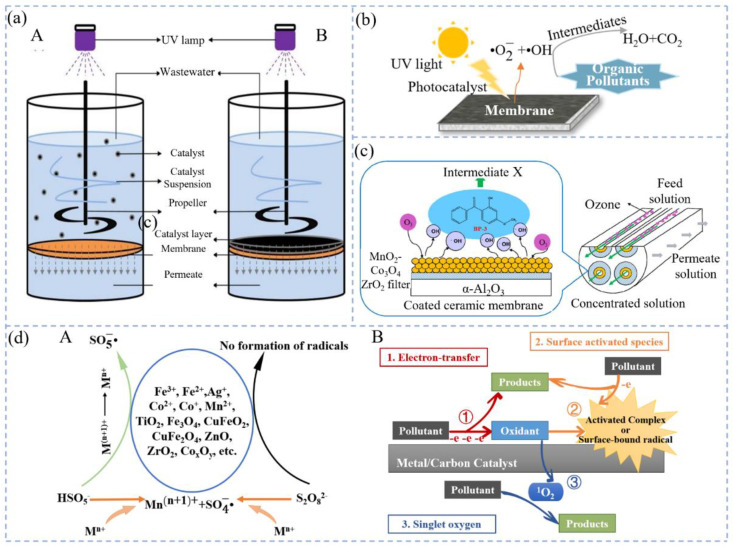
(**a**) Two different states of existence of photocatalysts in PMR: A, suspended on the membrane and B, loaded on the membrane [125]; (**b**) photocatalytic mechanism diagram of catalytic CM [133]; (**c**) schematic diagram of MnO_2_-Co_3_O_4_-loaded CM coupled with ozonation degradation of BP-3 [135]; (**d**) schematic diagram of mechanism activation of PS: A, schematic diagram of free radical mechanism for metal oxide activation of PS and B, schematic diagram of the non-radical mechanism of PS-activated catalytic oxidation of organic pollutants.

Photocatalysts can be divided into photocatalysts suspended in solution and photocatalysts fixed on a membrane (Figure 3a) [125]. In coupled systems, suspended photocatalysts are recovered by membrane energy separation, but may clog the membrane surface, resulting in decreasing permeate flux over time and increasing operating costs [126,127]. The preparation of photocatalytic membranes by immobilizing photocatalysts on membranes enables timely degradation of pollutants accumulated on the membrane surface and increases the treatment efficiency of the membrane unit [128,129,130]. UV irradiation can reduce the contact angle between the catalytic film and water, making the catalytic film super hydrophilic [131]. Under the irradiation of UV light, the electrons on the catalyst surface transition from the valence band to the conduction band, ultimately forming photogenerated electron hole pairs [132]. Photogenerated holes react with H_2_O to generate •OH (Figure 3b) [133,134]. These active free radicals react with organic pollutants adsorbed onto the surface of the catalytic membrane.

The photocatalysts that can be loaded onto the membrane through various manufacturing methods are divided into precious metals, metal oxides, and non-metal oxides (Table 2) [136,137,138,139,140,141,142,143,144]. TiO_2_ is the most widely used catalyst in membrane filtration-coupled photocatalysis, with a high surface area, photochemical stability, and excellent excited state lifetime [136,139,140,141,145]. Choi et al. [146] prepared nanostructured TiO_2_/Al_2_O_3_ composite membranes with a water permeability coefficient of 6.71 L m^−2^·h^−1^·bar^−1^ Under UV irradiation, it can decompose methylene blue dye and creatinine. Taking precious metal Pt as an auxiliary catalyst, Kumakiri et al. [147] loaded Pt onto CM with deposited TiO_2_. Under UV irradiation, the deposition of Pt increased the rate of formic acid oxidation by about 2–5 times. The Pt-TiO_2_ catalytic membrane avoided Pt oxidation and allowed it to remain in a metallic state even in the presence of reactive oxygen species. Compared with pure Pt catalytic membranes, Pt-TiO_2_ catalytic membranes have a longer lifespan. The bandgap width of TiO_2_ is relatively large (3.0–3.2 eV), and it can only absorb UV light. Doping TiO_2_ catalytic membrane with impurities can expand its light absorption range to the visible light range [140,141,144]. Liu et al. [137] used sol-gel and spray pyrolysis methods to grow N-TiO_2_ membranes on FTO glass, and deposited P-Cu_2_O onto them by a hydrothermal method. The surface of the Cu_2_O/TiO_2_ thin membrane was composed of a network and large grains, which had stronger optical absorption ability than pure TiO_2_ thin membrane. Membrane filtration coupled with a photocatalytic oxidation system can also kill bacteria, and Ag particles enhance the antibacterial effect of photocatalytic membranes [143].

Photocatalytic membranes are used to treat low-concentration pollutants [148]. Babu et al. [149] prepared a CuO-TiO_2_ photocatalytic membrane for the degradation of methyl orange dye. When the initial substrate concentration was between 0.01 mM and 0.04 mM, the degradation rate of the dye was highest at 0.01 mM. As the concentration of pollutants increases, the solution becomes opaque, and the pollutants themselves absorb light, thereby reducing the efficiency of the photocatalytic process [150]. In the actual wastewater treatment process, there are suspended substances with deep chromaticity and high turbidity, which reduce the transparency of the wastewater. The light intensity transmitted to the catalytic membrane is weakened, which has a negative impact on the photocatalytic efficiency [24]. Additional energy is required to achieve artificial UV irradiation, which adds significantly to operating costs. However, the pollutant removal efficiency is greatly reduced when using sunlight [19]. In the future, it is necessary to continue developing materials that can expand the range of light absorption. How to minimize the loss of light transmission to catalytic membranes is a hot research direction. In future experimental research, it is necessary to prepare an inorganic catalytic membrane that utilizes light energy more efficiently and has a wider range of light absorption.

### 3.2. IMF Coupled with Ozonation System 

The redox potential of O_3_ is 2.07 V, which allows oxidative decomposition of most of the organic pollutants in the water [151,152]. However, due to the unfavorable self-decomposition of O_3_ under environmental conditions, the utilization rate of O_3_ is low, resulting in low free radical yield. The reaction rate between O_3_ and organic compounds in the liquid phase is only 1.0 × 10^3^ M^−1^s^−1^, which limits its wider application [153]. The types of catalysts used to catalyze ozonation are divided into homogeneous and non-homogeneous types. Homogeneous catalysts are generally transition metal ions, which are difficult to separate from the effluent and easily cause secondary pollution [154]. Non-homogeneous catalysts are mainly classified as metal oxides (TiO_2_, MnO_2_, F_e2_O_3_, CeO_2_, etc.), noble metals, or activated carbon thereof, etc. (Table 3) [152,155,156,157,158,159,160,161,162,163,164]. Most of the non-homogeneous catalysts can be loaded onto the membrane to prepare a catalytic membrane [165,166]. O_3_ is activated by the catalyst on the surface of the catalytic membrane to generate •OH, and organic pollutants are efficiently degraded/mineralized into harmless inorganic compounds [167,168].

Compared with Mn CCM, Ce-CCM exhibits stronger mineralization ability and more effective O_3_ utilization during the mixing process. Li et al. [163] used a sol-gel-assisted impregnation method to prepare Mn-CCM and Ce-CCM, respectively. Under similar operating conditions, the specific O_3_(aq) of the mixing process of Ce-CCM consumption was 2.1 mg O_3_(aq) mg^−1^ TOC compared to 8.0 mg O_3_(aq) mg^−1^ TOC for Mn-CCM. Guo et al. [134] used a coupled system of IMF and catalytic ozonation to remove BP-3. The removal rate of BP-3 was 51.6% in membrane filtration alone, and only 47.4% in direct ozonation; the removal rate increased to 74.8%in MnO_2_-Co_3_O_4_@CM coupled with O_3_ (Figure 3c). The ion-leaching concentration was low (C_Mn_ < 2 mg/L). Under most conditions, IMF coupled with ozonation has a low utilization rate of O_3_ and sometimes produces disinfection byproducts (DBPs) that are more toxic than the original pollutants, which increases the harm to the environment. New catalytic membranes that can be developed in combination with ozonation can increase O_3_ utilization and pollutant mineralization, reduce DBP production, and reduce effluent toxicity [31,169]. Ozone can remove membrane fouling and reduce filtration resistance [170]. Making ozone purification applied to membrane cleaning research has become a hot topic in recent years. In the pilot system [164], the TMP increased to 35.9 kPa after a filtration time of 10 h only with Al_2_O_3_ membrane filtration. Under the same conditions, the catalytic TMP of the Mn-Al membrane increased to 24.2 kPa. When the ozone dosage increased to 5 mg/L, the TMP growth decreased to 4.6 kPa.

### 3.3. IMF Coupled with Persulfate Oxidation System

Peroxide (-O-O-) is present in persulfate molecules that have strong oxidizing properties. Its standard electrode potential is 2.5–3.1 V, exceeding O_3_ (+2.07 V). The $SO_{4}^{-}•$ generated by activating PS has a high potential and can rapidly oxidize organic pollutants like HO• [171,172]. Thermal energy, UV and visible light, alkali, transition metals, etc. can effectively activate persulfate (PS) to generate $SO_{4}^{-}•$ and HO• [173,174,175]. Transition metals and their oxides can be loaded onto the membranes to prepare catalytic membranes, which are coupled with persulfate oxidation for use (Table 4) [176,177,178,179,180,181,182,183,184,185]. There are usually three mechanisms to control PS-based AOPs for the degradation of organic pollutants: (i) by free radicals (in most cases, $SO_{4}^{-}•$, •OH and $O_{2}^{-}•$); (ii) non-free radicals (^1^O_2_); and (iii) direct oxidation (Figure 3d) [135,182,186,187,188].

Al_2_O_3_-based and SiO_2_-based membranes have good thermal stability [2]. Shan et al. [189] prepared a flexible copper droplet carbon/SiO_2_ nanofiber membrane (Cu@C/SiO_2_ NFM). The membrane produced could remove 95% of tetracycline hydrochloride (TCH) from wastewater. Bao et al. [178] prepared a CoFe_2_O_4_@CM catalytic membrane with a pure water permeate flux of 3000 L m^−2^·h^−1^·bar^−1^. They [177] also prepared Co_3_O_4_ nanocatalyst-functionalized Al_2_O_3_ CMs (CoFCM) with a honeycomb structure by using an optimized surface-nucleated heterogeneous growth of zeolitic imidazolate framework (ZIF-67) method in the same period. CoFCM and CoFe_2_O_4_@CM catalyzed the removal of over 90% SMX by persulfate. Zhao et al. [190] found that the CuFe-CM/PMS system can degrade organic matter into stable, low-molecular-weight unsaturated bonds, and the products aggregate with each other, thereby reducing the irreversible fouling of the membrane. Wu et al. [191] prepared a MnO_2_/Al_2_O_3_ membrane that retained a high degradation rate even after six repetitions. Quenching experiments with NaN_3_ and ethanol confirmed that the free radical pathway was not the dominant route of degradation. Meanwhile, studies [180] have demonstrated that Mn CMs/PMS has a non-free radical mechanism for the degradation of EDCs. The oxidation-based process of ^1^O_2_ exhibits special selectivity for the removal of phenols and bisphenols.

Carbon material membranes have great potential in SR-AOPs due to their ability to activate persulfate. Song et al. [192] designed a novel coal-based carbon catalytic membrane that could activate PDS to degrade phenol. Synergistic effects of free and non-free radicals were found to co-exist in the study, but the effect percentage was not clear. Low TMP represents energy saving and cost reduction in the filtration process. It has been found that rGO promotes TMP reduction, and GO-based composite membranes exhibit considerable antifouling ability in situ catalytic oxidation [185]. C=O groups are the active sites for CS carbon-catalyzed PMS activation, and C-OH groups are the active sites for PS activation in mesoporous carbon [193,194]. However, the intrinsic interactions between the oxidizer and the catalyst in the non-radical pathway are still unclear and need to be investigated further. Further research on the mechanism of persulfate oxidation may be a hot direction in the future, which is crucial for improving the performance of mixing processes.

### 3.4. IMF Coupled with Fenton and Composite Fenton Oxidation Systems 

The essence of catalytic wet peroxide oxidation (CWPO) technology is to activate H_2_O_2_ to generate HO• for the degradation of organic compounds [195,196]. Current methods regarding H_2_O_2_ include Fenton, electro-Fenton (EF), and photo-Fenton (PF) [135].

#### 3.4.1. IMF Coupled with Fenton Oxidation Systems

The pH range of Fenton technology alone is narrow (2.8~3.0), and adding a large amount of iron salts will inevitably lead to the formation of iron sludge in the wastewater treatment process. The generated iron sludge needs to be collected and processed, which increases operating costs [197,198]. Traditional solid metal oxidants are prone to agglomeration and have fewer exposed active sites [199]. The catalytic membrane-coupled Fenton system can increase the metal active sites loaded onto the surface or embedded in the membrane channels, promote the decomposition of H_2_O_2_, and enhance oxidation performance (Table 5) [200,201,202,203,204,205,206,207,208,209]. Wang et al. [201] and Shi et al. [202] immobilized MnO_2_ and CuFe_2_O_4_ on SiO_2_ nanofiber membranes (Mn-SiO_2_ membrane and CuFe-SiO_2_ membrane). Both membranes mentioned above could activate H_2_O_2_ over a wider pH range. MOF catalysts showed excellent catalytic activity with poor stability in the CWPO process [210,211]. Jiang et al. [204] optimized the preparation of UiO-66@Al_2_O_3_ ceramic tube membranes (UiO-66CT) with the Cu or Mn added. The removal rate of phenol was >90% after five cycles. This study provides a promising strategy to address the instability of MOF catalysts during the CWPO.

#### 3.4.2. IMF Coupled with Composite Fenton Oxidation Systems

**Figure 4:**
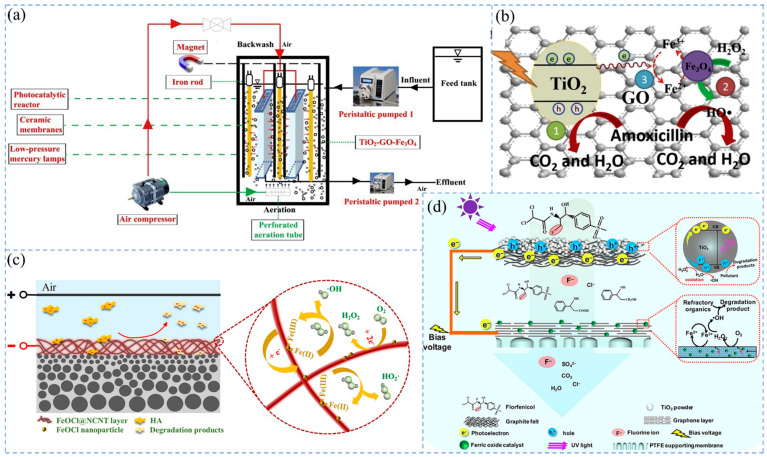
Mechanism of membrane filtration coupled with composite Fenton oxidation: (**a**) SMSMPR SMSMPR process flow diagram [206], (**b**) mechanism of TiO_2_-GO-Fe_3_O_4_ composite membrane-coupled PF degradation AMX [206], (**c**) proposed EF catalytic mechanism of the FeOCl@NCNT/CM during the EF catalytic filtration [213], (**d**) schematic representation of the reactive oxygen species mediated mechanisms for florfenicol degradation in the PEC/EF filtration process [214].

The PF process simply adds sunlight or UV with appropriate wavelengths to the Fenton process [212]. Li et al. [206] combined GO and highly magnetic Fe_3_O_4_ with TiO_2_ components (TiO_2_-GO-Fe_3_O_4_) loaded onto flat CMs of SMSMPR (Figure 4a). This system destroyed -O-O- to generate HO- by UV irradiation and accelerated the reaction (Figure 4b). Degradation of amoxicillin (AMX) showed only 22.3% degradation of AMX under dark conditions and 88.5% degradation of AMX by under UV irradiation.

When membrane filtration is coupled with Fenton technology with an applied current, it can catalyze at a higher and wider pH range [215,216,217]. H_2_O_2_ in EF technology can be produced by double electron reduction of dissolved oxygen on the cathode [5,218]. Due to the insulation of CM, carbon material membranes are commonly used for IMF coupling with EF. Yang et al. [219] used CNTs and porous carbon (PC) to prepare PC-CNT hollow fiber membranes and found that the efficiency of PC-CNT membranes and the coupled EF process for treating phenol was about 9.7 times higher than that of a single-membrane separation. Layered metal halide oxides have a unique two-dimensional intercalation structure and excellent electrical conductivity. Li et al. [209] developed a FeOCl functionalized reactive porous CNT electrochemical reaction filter to degrade TC. This system showed enhanced oxidation kinetics compared with conventional batch reactors. Nano FeOCl significantly promoted the production of •OH through the highly efficient cycling of Fe^3+^/Fe^2+^ (Figure 4c) [213]. Zhang et al. [213] prepared a FeOCl@NCNT/CM to control membrane fouling caused by HA. The FeOCl@NCNT/CM exhibited excellent fouling resistance. At an applied voltage of −2.2 V, the water flux of the membrane was 1.79 times higher than that without voltage. The catalytic membrane/EF system has excellent performance, but the cathode material has strict requirements for hydrophilicity, porosity, and surface area [64,220]. The external power supply may accelerate the corrosion of the membrane material and shorten the service life. To put the catalytic membrane/EF system into practical applications, it is necessary to design better composite membranes for continuous simulation experiments.

In addition, Jiang et al. [214] proposed a novel UV-driven electro-Fenton catalytic membrane (UV-EFCM) filtration system for the first time (Figure 4d). UV-EFCM used a TiO_2_-modified graphite felt filter (TiO_2_/GF) as a photoanode to treat a low concentration of florfenicol (14 μM), achieving almost complete degradation and high mineralization (78.4 ± 9.1%). This integrated system utilizes photovoltaic electronics to save energy requirements and is expected to be a hot research direction in the future.

### 3.5. Comparison of Different IMF Coupled with AOPs

In Section 3.1, Section 3.2, Section 3.3 and Section 3.4, the relevant applications of IMF coupled with the AOP process were introduced and discussed. We summarized the advantages and disadvantages of the above IMF coupled with photocatalytic oxidation, IMF coupled with ozonation, IMF coupled with persulfate oxidation, and IMF coupled with Fenton oxidation (Table 6). The combination of electrochemistry and membrane filtration is another advanced wastewater technology [221]. In the presence of an electric field, various types of fouling with different charging characteristics can migrate outward from the membrane surface by electrostatic repulsion or electrophoresis, or even be eliminated electrochemically by oxidation or reduction reactions [222]. CBMs have good conductivity and are often coupled with electrocatalysis. This system improves the fouling ability of the membrane and has a high removal rate for pollutants with molecular sizes smaller than the membrane pore size [223,224]. Li et al. [225] prepared dynamic electrodeposited CuO/carbon membranes by depositing CuO nanoparticles uniformly on the surface and pore wall of a coal-based carbon membrane. The removal rates of rhodamine B (RhB) and COD by this membrane coupled with electrocatalysis were 99.96% and 71.82%, which were 20 times and 1.8 times higher than those of the original carbon membrane and conventional electrodeposited CuO/carbon membrane, respectively. However, membrane filtration coupled with electrocatalysis increases energy consumption and cost accordingly. The total process energy consumption needs to be carefully considered to verify the sustainability of using additional electric fields. More research on coupling electrochemistry with membrane filtration can be conducted in the future.

## 4. Challenges and Prospects

Membrane technology is a cost-effective and simple process widely used in different applications in the separation industry, especially in water treatment and water treatment applications. This review focused on different types of inorganic membranes in membrane filtration for water treatment applications, including CMs and CBMs, which have been a hotspot of interest in recent years. Compared with organic membranes, inorganic membranes are superior in terms of permeate flux, mechanical stability, and thermal stability. Inorganic membranes are more competitive, so the research on inorganic membrane separation technology in wastewater treatment has been gradually increasing. So far, most of the reported CMs and CBMs have superior performance, but the manufacturing costs are high and not as economical as polymer membranes. The preparation of catalytic membranes can change the membrane performance to reduce membrane contamination. It is known that AOP processes are effective, but they can cause accelerated formation of degradation products. The use of both AOPs and membranes can be a good solution and effective in combating transformation products. The coupling of IMF with AOPs can not only efficiently separate pollutants, but also directly degrade organic pollutants in situ and improve the effluent water quality. However, the coupling process is susceptible to the influence of other factors, rarely used in practice. And for the additional oxidant and energy cannot be fully utilized.

There are still problems in the development and production of IMF and IMF coupled with AOPs, mainly including the following six aspects.

(1) Higher cost of inorganic membranes. Despite the longer service life of inorganic membranes, both ceramic and carbon material membranes have high manufacturing costs, which limits the practical application of inorganic membranes. For CM, cost-effective natural materials such as kaolin, pyroxene, and dolomite can be used as raw materials [230,231]. Carbon nanomaterials can be used in combination with other materials to reduce the proportion of carbon materials while ensuring membrane performance [14,232]. All of these can be used as a means to reduce costs. The use of natural materials will inevitably bring other components to inorganic membranes, leading to membrane defects [14]. In the future, it will be necessary to optimize the preparation method of inorganic membranes and adjust the material ratios to create more cost-effective membrane materials.
(2) Membrane fouling affects the service life of the membrane. Some IMFs are prone to membrane fouling. Reversible membrane fouling could be removed by cleaning the membrane, but excessive cleaning times will inevitably reduce the service life of the membrane. Irreversible membrane fouling cannot be eliminated [115] and will inevitably affect the permeation flux of the membrane. The membrane can be modified by loading other materials to alter its surface properties [64,233], such as roughness, hydrophilicity, stability, membrane surface charge, etc. Currently, many materials have been applied to the preparation of catalytic membranes. Materials that can provide high permeability and selectivity as well as low energy consumption, will be one of the most effective materials for doping membranes.
(3) Low catalytic membrane recovery. The use of O_3_ or the use of tiny electric field cleaning can effectively recover membranes during wastewater treatment [120,133,234]. But research is only at the laboratory stage and practical water treatment applications are not always feasible. The toxic waste generated after cleaning requires specialized treatment and disposal. Therefore, new environmentally friendly cleaning agents should be prepared to achieve sustainable long-term membrane operation.
(4) The application examples of IMF-AOPs are scarce [31]. In the literature on using membrane filtration coupling with the AOP process to remove pollutants, most of them are laboratory-simulated water samples instead of real wastewater samples. Real wastewater environments are very complex. In order to better apply catalytic membranes to long-term real-world processes, the stability and effectiveness of these catalytic processes need to be explored using real water quality.
(5) In the future, it is necessary to further improve the performance of the IMF coupled with -AOPs system through optimal design. The utilization rate of oxidizer and the removal efficiency of pollutant can be improved in the coupling system while maintaining low energy consumption. And it is best to minimize the membrane pollution and improve the reuse performance of the membrane [197]. CBMs have high electrical conductivity [64,235], and catalytic CMs with electrical conductivity can reduce internal resistance due to their large thickness. The application of inorganic membranes to electrocatalysis is a future direction [220,236]. On the other hand, multifunctional catalytic membranes can be developed in the future, considering the trade-off between processing efficiency and energy consumption. The developed catalytic membranes can be applied to the removal of different pollutants under one or even multiple AOP systems.
(6) The mechanism of interaction between membranes and contaminants in the IMF coupled with AOPs process has not been clearly described so far. The current mechanism of the membrane filtration and AOP coupling process is based on the removal of pollutants by AOPs. The mechanisms of synergism and interaction between contaminant degradation intermediates and membrane skeleton surfaces remain unclear. Elucidating the dynamic application of inorganic membranes in contaminant degradation can provide a clearer understanding of the membrane fouling mechanism, thus effectively preventing membrane contamination and improving membrane lifetime.

## 5. Conclusions

This review aimed to explore the research progress of IMF technology in wastewater treatment. According to a systematic review of existing literature, inorganic membranes are superior to polymer membranes in terms of service life, permeation flux, and cleaning efficiency, making them more competitive. The research on IMF in wastewater treatment has been gradually increasing. CMs and CBMs are currently the most popular inorganic membranes for research. Membrane filtration technology achieves wastewater purification through a screening mechanism, and the accumulated pollutants require subsequent treatment. The properties of inorganic membrane surfaces can be changed by membrane modification to provide superior separation capability. At present, coupling IMF with AOPs (including IMF–photocatalysis oxidation, IMF–ozonation, IMF–persulfate oxidation, and IMF–Fenton oxidation) has received widespread attention as a novel catalytic inorganic membrane filtration process. These combined processes can degrade pollutants while separating them, but further exploration through detailed technical and economic analysis is needed for a comprehensive application. With more and more emerging ideas and strategies to improve processing techniques and minimize defects, inorganic membranes have broad potential in practical applications. In the future, more efforts should be made to reduce the production cost and practical application of ceramic membranes.

## Figures and Tables

**Table 1:** Effect of different strategies on membrane fouling mitigation during wastewater treatment.

Membrane Properties	Operating Conditions	Strategy	Fouling Mitigation Effect	Comments	Ref.
Flat ceramic membrane (200 nm)	HRT: 0.5 h, Flux: 30 LMH, MLSS: 1 g/L, O_3_ and NaClO: 0~5 mg/L	Chemical cleaning	O_3_ effectively degraded larger biopolymers to low molecular weight substances.	O_3_ cleaning produced less TOCl, which was more environmental.	[117]
Flat ceramic membrane	HA: 50 ± 1 g/L, Air flow rate: 5.83 × 10^−6^~7.50 × 10^−6^ m^3^/s, TMP: 413.7 kPa	Air NBs cleaning	The permeation flux of CM basically recovered to 99%.	NBs successfully unclogged the pores of the membrane.	[114]
ZrO_2_/SiC UF membrane	Cross flow: 1527 L/h, HRT: 80 min, TMP: 1 bar	Dip coating a SiC support with a ZrO_2_ slurry	The test of olive oil/water emulsion removed 99.91% of oil without fouling.	Long-term corrosion tests did not cause change in morphology.	[107]
TiO_2_ ceramic membrane	HRT: 3 h, pH: 6~9, Temp.: 20 ± 1 °C, Flux: 62 LMH	SWRO pretreatment	SWRO pretreatment limited biofouling in RO by inducing phosphate limitation.	Phosphate removal was up to 87%.	[118]
Al_2_O_3_ UF membrane	Flux: 90 LMH, Temp.: 22~25 °C, Ferrate: 0.15 mM	Integration of ferrate (VI) pretreatment	Cake layer scaling decreased significantly, TMP decreased by 81.8%.	Cake layer became more porous with increase in ferrate.	[119]
Ceramic UF membrane	pH: 7.0 ± 0.1, TMP: 50 kPa, Fe(II): 15 or 50 μΜ, PMS: 15 or 50 μΜ, Temp.: 25 ± 1 °C	Fe(II)/PMS pretreatment	The rate of reversible and irreversible fouling was reduced by 83.5% and 96.5%.	Low dose of Fe(II)/PMS aggravated membrane fouling caused by BSA.	[120]
CNTs-Al_2_O_3_ composite membrane	Electric field: >3 × 10^3^ kV/m, Flux: 75 LMH, Temp.: 25 ± 2 °C, TMP: 1.5 bar	Coupled with ultrasound	The recovered water permeance of the membrane which coupled with ultrasound.	Microcurrents or vibrations resulted in reduced concentration polarization.	[121]

TOCl: total organochlorine, NBs: nano bubbles.

**Table 2:** Summary of recent works of IMF coupled with photocatalytic oxidation in pollutant removal.

Catalyst	Support Material/Membrane	Exp. Conditions	Efficiency	Comments	Ref.
TiO_2_	Tubular Al_2_O_3_ UF membrane	UV intensity: 1.54 W/m^2^, TOC: 5 ± 0.5 mg/dm^3^, CFV: 3~6 m/s, TMP: 0.1 MPa, HA: 5 ± 0.5 mg TOC/dm^3^	Removal rate: >95%, Mineralization rate: 70%.	Pure water flux: 367 dm^3^/(m^2^·h). Stable operation for 400 h.	[136]
Cu_2_O/TiO_2_	FTO conducting glass	Mercury lamp: 125 W, Light intensity: 1.8 mW/cm^2^, MB: 10 ppm, pH: 4 or 8	MB: 80%.	Cu_2_O/TiO_2_ film could utilize visible light.	[137]
SiO_2_/TiO_2_ nanorods/nanotubes	Alumina template membrane (200 nm)	Flow velocity: 12.7 L/h, Temp: 298 K, UV: 400 μW/cm^2^, pH: 3.5	SDBS: 89%.	The composite membrane had better removal.	[138]
rGO/TiO_2_ and N-TiO_2_-10	Tubular γ-Al_2_O_3_ UF membrane	UV intensity: 2.1, 7.2 mW/cm^2^, Flow rate: 1.5 mL/min, MO: 6.4 mg/L, MB: 2 mg/L	MB: 63%.	MB removal was superior to MO.	[139]
N-TiO_2_	Ceramic UF membrane	Xenon lamp intensity: 300 W, Water flow rate: 4 L/min, TMP: 0.4 MPa, pH: 7, Dye: 10 mg/L	The Retention rate: 99%. Water flux: 20 LMH.	The visible light effect was poor. Could be reused 7 times.	[140]
N-TiO_2_	Commercial α-Al_2_O_3_ UF membrane	UV intensity: 712.3 W/m^2^, CBZ: 4.24 × 10^−3^ mM, pH: 7	CBZ: 90%	N-doped composite films utilized sunlight more efficiently.	[141]
CdS	PAA	Visible light intensity: 2.53 mW/cm^2^, CO_2_ flow velocity: 3 cm^3^/min	45.4%	-	[142]
AgNCs	g-C_3_N_4_/NF hybrid membrane	Xenon lamp intensity: 420 W, AgNCs: 4~5 nm, RhB: 10 mg/L	RhB: 86%.	Maintained good stability after 5 cycles.	[143]
Fe-doped TiO_2_	rGO	pH: 6, RhB: 20 mg/L, TOC: 930 mg/L, COD: 1550 mg/L	The removal of TOC: 66.7%. The removal of COD: 59.1%.	The removal rate decreased slightly (about 12%) after 5 cycles.	[144]

CFV: cross-flow velocity, MO: methyl orange, MB: methylene blue, SDBS: sodium dodecyl benzene sulfonate, CBZ: carbamazepine, PAA: porous aluminum anodizing film, AgNCs: Ag nanocrystals.

**Table 3:** Summary of recent works of IMF coupled with ozonation in pollutant removal.

Catalyst(s)	Membrane	Exp. Conditions	Efficiency			Comments	Ref.
			Membrane	O_3_	Coupling		
–	Tubular Al_2_O_3_ UF membrane (pore size: 0.5 μm)	O_3_: 9.5 g/m^3^, O_3_ flow rate: 0.2 L/min, Temp: 22.5 °C, TOC: 11.8 mg/L	-	-	15% reduction in flux.	Osmotic flux increased with increasing O_3_ concentration.	[152]
Fe_2_O_3_ or TiO_2_ or MnO_2_	CM (MWCO: 5 kDa)	O_3_ flow rate: 10 mL/min, O_3_: 10 g/m^3^, TMP: 1.9~2.2 bar, TOC: 10.4 mg/L	-	-	THM: 39%, HAA: 55%.	The fouling behavior: Fe_2_O_3_ > TiO_2_ > MnO_2_	[155]
Fe_2_O_3_	γ-Al_2_O_3_ UF membrane	O_3_: 10 mg/L, pCBA: 3.4 mg/L, pH: 7, TMP: 80 kPa Water flux: 0.02 L/min	8%	28%	46%	Effective in controlling NOM pollution.	[156]
TiMn_2_O_3_	CM	O_3_: 2.5 mg/L, COD_Cr_: 100 ± 20 mg/L, SS: 20 ± 5 mg/L, *E. coli*: >2.4 × 10^6^ MPN/L	65%	60%	Reduced membrane flux: 53%.	Chroma, SS, and *E. coli* were entirely removed.	[157]
Three types of MnO_2_	TiO_2_ membrane	O_3_: 0.5 mg/L, Water flux: 1.0 mL/min, TMP: 100 kPa, SA: 1.0 g/L, p-CNB: 100 μg/L	15%	41.4%	(a) 51.7%, (b) 61.5%, (c) 68%.	S-MnO_2_ has the best ozonation effect.	[158]
Ti–Mn/TiO_2_	Tubular γ-Al_2_O_3_ membrane	O_3_ flow rate: 52 mg/min, TMP: 2.22~2.30 bar, Water flux: 1.04 ± 0.04 m/s	55%.	-	SS removal rate: 100%.	Short start-up times and stable running conditions.	[159]
CuMn_2_O_4_	Tubular CM	O_3_: 1.0 mg/L, BP-3: 2 mg/L, pH: 7.2	51.6%	47.4%	71.2%	Decreased the UV_254_ and DOC of effluent.	[160]
CeO_x_/MnO_x_	Tubular α-Al_2_O_3_ membrane	O_3_: 500 mL/min, Feed flux: 20 mL/min, HRT: 13.7 S, BPA/BTZ: 3 mg/L,	BPA: 55%	-	BPA: 84%, BTZ: 57%	Higher ozone utilization of Ce-CCM.	[161]
MnO_x_	CM	Ozone: 5 mg/L, Water flux: 80 LMH, HRT: 4 h	31.2%	-	39.5%	-	[162]
MgO, CeO_x_, and MnO_2_	Flat-sheet CM	Water flux: 62.5 LMH, 4BS: 12 mg/L, HRT: 30 s, O_3_ flow rate: 3.3 g/h	-	38%	Mg-Ce: 85%, Mg-Mn: 88%.	Effective reduction of membrane fouling.	[163]
Fe_2_O_3_	Tubular CM	Flow rate: 69 L/h, O_3_ flow rate: 20 L/h, pH: 8.5, IBU:10 mg/L	76%	-	99%	The ozone-NF system reduced the toxicity of pollutants.	[164]

pCBA: para-chlorobenzoic acid, p-CNB: penta chloronitro benzene, BP-3: benzophenone-3, BPA: bisphenol A, IBU: ibuprofen, BTZ: benzotriazole.

**Table 4:** Summary of recent works of IMF coupled with persulfate oxidation in pollutant removal.

Catalyst(s)	Membrane	Exp. Conditions	Efficiency	Comments	Ref.
CuO	α-MnO_2_ nanowire membrane	Flow rate: 20 mL/min, pH: 7.4, PMS: 1.0 mM, MB: 0.1 mM	MB: >99%.	It also had a high degradation effect on other kinds of dyes.	[176]
Co_3_O_4_	Al_2_O_3_ CM	PMS: 0.1 g/L, TMP: 0.07 bar, SMX: 10 mg/L, pH: 5	SMX: 90%.	Maintained 95% of initial flow rate after 3 cycles.	[177]
CoFe_2_O_4_	Porous Al_2_O_3_-based filter substrate	PMS: 0.1 g/L, SMX: 10 mg/L, Temp: indoor temperature, P: 0.09 bar, pH: 5	SMX: 98%.	It was well tolerated in a wide pH range (3–11) and different anions.	[178]
CuO	CHFMs	PMS: 0.5 mM, BPA: 10 mg/L, T: 25 °C, pH: 7	BPA: >98.5%.	^1^O_2_ dominated non-radical pathway.	[179]
-	Mn_2_O_3_-Al_2_O_3_ membrane	EDCs: 0.1 mg/L, PMS: 0.3 mM, Flux: 60 LMH	Trace EDCs: >95%.	Reduced manganese ion leaching.	[180]
Co_3_O_4_	Al_2_O_3_ membrane	PMS: 4 mM, TMP: 2 bar, CFV: 5 mL/s, HBA: 20 ppm, pH: 7	HBA: >95%.	Good water flux even at HA concentration of 200 ppm.	[181]
CuCo_2_O_4_	α-Al_2_O_3_ membrane	BPA: 30 mg/L, pH: 7.0, PMS: 2 mM, Flux: 650 LMH	BPA: >92.1%.	Ion leaching of Co and Cu within a safe range.	[182]
NiCo_2_S_4_	CS	Nim: 5 mg/L, PDS: 0.4 g/L, Temp: 25 °C, V: 50 mL	Nim: >94%.	The degradation rate was 81% when repeated 6 times.	[183]
Ni-Co	NCNTs	PMS: 0.65 mM, IBP: 20 mg/L, Temp: 25 °C, Catalyst: 0.05 g/L	IBP: 98%.	Metal leaching was extremely low.	[184]
rGO	CNTs	PS: 5 mM, Flow rate: 1.0 mL/min, SMX: 500 μg/L, Temp.: 25 ± 2 °C	SMX: 98%.	The optimal C/O ratio was estimated at 2.9.	[185]

PMS: peroxymonosulfate, SMX: sulfamethoxazole, CS: carbon sphere, Nim: nimesulide, NCNTs: N-doped carbon nanotubes, IBP: ibuprofen, CHFM: ceramic hollow fiber membrane, EDCs: endocrine disrupting compounds, HBA: 4-hydroxybenzoic acid.

**Table 5:** Summary of recent works of IMF coupled with Fenton, electro-Fenton, and photo-Fenton oxidation in pollutant removal.

AOPs	Catalyst(s)	Membrane	Exp. Conditions	Efficiency	Comments	Ref.
Fenton	–	Cu-Al_2_O_3_ fibrous membrane	BPA: 20 mg/L, pH: 7 H_2_O_2_: 12 mM, Water flux: 0.3 mL/min,	BPA: >87%	High Fenton catalytic activity at neutral pH.	[200]
	MnO_2_	SNM	MB: 10 mg/L, Temp: 23 ± 3 °C, P: 5 kPa, pH: 6	Fenton: 6%, SiO_2_/Fenton: 9%, Mn-NFM/Fenton: 90%.	High degradation properties after five cycles.	[201]
	CuFe_2_O_4_	SNM	MB: 10 mg/L, pH: 6	MB: 96%.	The degradation degree was 85.7%.	[202]
	Fe_3_O_4_	Tubular α-Al_2_O_3_ membrane	H_2_O_2_: 8.7 mg/L, pH: 3.0 Water flux: 0.5 L/min, DCF: 282.4 μg/L,	Removal rate: 65.1% Mineralization rate: 47.9%.	Wide pH range, low ferrous ion leaching.	[203]
	Cu-UiO-66 or Mn-UiO-66	Tubular Al_2_O_3_ membrane	Phenol: 100 mg/L, H_2_O_2_: 510 mg/L, V: 200 mL, T: 60 °C	Phenol: 99%.	Higher catalytic activity of the Cu-UiO-66@CM.	[204]
Photo-Fenton	M88A (Fe)	GO	MB: 10 mg/L, H_2_O_2_: 10 mM, Light intensity: 10^4^ mW/cm^2^	Dark: 42.1%, PF: 98.8%.	The MB degradation rate was 97.87% after 12 cycles.	[205]
	TiO_2_-GO-Fe_3_O_4_	Flat CM	H_2_O_2_: 20mM, AMX: 20 mg/L, Flux: 100 mL/min	AMX: 88.5%.	Composites exhibited PF catalytic property.	[206]
Electro-Fenton	GO	PTFE	Florfenicol: 1 mg/L, H_2_O_2_: 5 mg/L, Potential: −0.6 V, Na_2_SO_4_: 20 mM	Single filtration: 27%, EF: 90%.	The process can be used for advanced water purification.	[207]
	Graphene	SS membrane	Potential: −0.5 V, PCM: 0.1 mM, I: 170 mA, pH: 3	Mineralized current efficiency values increased by 165%	Remained stable for 3 cycles.	[208]
	FeOCl	CNT	Potential: −0.8 V, TC: 0.04 mM, pH: 6.5, Na_2_SO_4_: 10 mM	TC: >95%.	Wider range of pH applications.	[209]

DCF: diclofenac, SNM: SiO_2_ nanofibrous membrane, AMX: amoxicillin, SS: stainless steel, PCM: paracetamol, TC: tetracycline.

**Table 6:** Comparison of different IMFs coupled with AOPs.

Coupling Process	Advantages	Disadvantages	References
IMF coupled with photocatalytic oxidation	Increases the hydrophilicity of the catalytic membrane. Mild reaction conditions. No additional chemical reagents are required. Easy to combine with other AOPs.	Requires illumination. Low efficiency of visible light utilization. Poor treatment of high suspended solids and high-turbidity wastewater.	[19,24,125,136,226]
IMF coupled with ozonation	Quickly oxidizes contaminants. Wider pH range.	Requires an ozone generator with high operating costs. Easy to generate DBP in the reaction process. Low ozone utilization rate.	[20,31,227]
IMF coupled with persulfate oxidation	Higher oxidation potential for greater oxidizing power. Cheaper persulfate oxidizer.	PS price is high. The remaining $SO_{4}^{-}•$ needs to be processed later. The mechanism of non-radical reactions is not yet clear.	[135,194,228]
IMF coupled with Fenton oxidation	Low cost. Convenient operation. Mild reaction conditions. Widely applicable.	More oxidant consumption and relatively weaker oxidation ability. Generate a large amount of iron sludge. Narrow pH range.	[21,135,229]
IMF coupled with composite Fenton oxidation	A wider range of pH applications. The amount of iron mud generated decreases. A decrease in iron loss. Improves the utilization rate of oxidants.	Requires lighting or electricity to increase energy consumption. The practical operation process requires higher requirements.	[209,215]

## Data Availability

Not applicable.
